# Circ_0000069 promotes the development of hepatocellular carcinoma by regulating CCL25

**DOI:** 10.1186/s12885-024-12594-y

**Published:** 2024-07-11

**Authors:** Junshao Zeng, Yi Feng, Liwen Lin, Huifeng Ye, Haoming Shen, Yifan Sun

**Affiliations:** 1 Department of Oncology, The Eighth Affiliated Hospital of Guangxi Medical University, Guigang City People’s Hospital, Guigang, Guangxi China; 2 Department of Clinical Laboratory, The Eighth Affiliated Hospital of Guangxi Medical University, Guigang City People’s Hospital, No. 1, Zhong Shan Road, Guigang, 537100 Guangxi China; 3 Department of Clinical Laboratory, Hunan Cancer Hospital &, The Affiliated Cancer Hospital of Xiangya School of Medicine, Central South University, Changsha, Hunan China

**Keywords:** Hepatocellular carcinoma, Circ_0000069, MAP2K1, CCL25, Progression

## Abstract

**Background:**

Hepatocellular carcinoma (HCC) is a leading cause of cancer-related deaths globally, influenced by aberrant circRNA expression. Investigating circRNA-miRNA-mRNA interactions can unveil underlying mechanisms of HCC and identify potential therapeutic targets.

**Methods:**

In this study, we conducted differential analyses of mRNAs, miRNAs, and circRNAs, and established their relationships using various databases such as miRanda, miRDB, and miTarBase. Additionally, functional enrichment and immune infiltration analyses were performed to evaluate the roles of key genes. We also conducted qPCR assays and western blotting (WB) to examine the expression levels of circRNA, CCL25, and MAP2K1 in both HCC cells and clinical samples. Furthermore, we utilized overexpression and knockdown techniques for circ_0000069 and conducted wound healing, transwell invasion assays, and a tumorigenesis experiment to assess the migratory and invasive abilities of HCC cells.

**Results:**

Our findings revealed significant differential expression of 612 upregulated genes and 1173 downregulated genes in HCC samples compared to normal liver tissue. Additionally, 429 upregulated circRNAs and 453 downregulated circRNAs were identified. Significantly, circ_0000069 exhibited upregulation in HCC tissues and cell lines. The overexpression of circ_0000069 notably increased the invasion and migration capacity of Huh7 cells, whereas the downregulation of circ_0000069 reduced this capability in HepG2 cells. Furthermore, this effect was counteracted by CCL25 silencing or overexpression, separately. Animal studies further confirmed that the overexpression of hsa_circ_0000069 facilitated tumor growth in xenografted nude mice, while the inhibition of CCL25 attenuated this effect.

**Conclusion:**

Circ_0000069 appears to promote HCC progression by regulating CCL25, suggesting that both circ_0000069 and CCL25 can serve as potential therapeutic targets.

**Supplementary Information:**

The online version contains supplementary material available at 10.1186/s12885-024-12594-y.

## Introduction

Hepatocellular carcinoma (HCC) remains the most common primary liver cancer globally, significantly contributing to cancer-related mortality with an estimated 780,000 deaths annually [1, 2]. Despite advancements in diagnostic methods and treatment strategies, the five-year survival rate for HCC patients is still disappointingly low [3, 4]. This has partly been explained by delays in HCC diagnosis and treatment [3]. Simultaneously, while molecular targeted therapies and chemotherapy are available for some HCC patients, only a small subset of patients respond to immunotherapy. Furthermore, the development of resistance complicates the clinical management of HCC [5]. Therefore, early diagnostic markers and treatment targets are urgently needed to improve the prognosis of HCC patients.

Traditionally, HCC research has focused on protein targets for developing new therapeutic strategies. Recently, there has been an increasing interest in non-coding RNA (ncRNA) as potential diagnostic markers and therapeutic targets for cancer [6, 7]. NcRNA components include microRNA (miRNA), long non-coding RNA (lncRNA) and circular RNA (circRNA) [8]. Recent studies have highlighted that specific miRNAs, such as miR-122 and miR-194/192 in liver cells, miR-223 in neutrophils, miR-29 in hepatic stellate cells, and miR-155 in immune cells, as well as exosomes, are crucial as regulators, biomarkers, and therapeutic targets in HCC [9]. Similarly, the highly regulated lncRNA, such as HULC, MALAT1, and HOTAIR, have been identified as potential therapeutic targets for HCC [10, 11]. The detection of HULC in the plasma, especially prevalent in patients with higher Edmondson grades or positive hepatitis B virus (HBV) status [12], indicates its potential as a non-invasive, valuable biomarker for diagnosing and prognosticating HCC. Newer research shows that circZFR acts as a competitive endogenous RNA by sponging microRNA-624-3p to regulate WEE1 expression, thereby promoting tumor cell proliferation, migration, and invasion [13].Therefore, the exploration of ncRNA as a source of diagnostic biomarkers and therapeutic targets for HCC holds promise for disease management.

CircRNA is a distinct type of RNA, characterized by a covalently closed loop structure without a 5' or 3' end, contributing to its stability and potential utility as a biomarker or therapeutic agent. CircRNAs are pivotal in regulating cellular functions and influencing protein synthesis, particularly in cancer research [14, 15]. Their role as miRNA sponges, affecting the expression of miRNA target genes, implicates them in the pathogenesis of various cancers, including HCC [16]. Studies have demonstrated the importance of circRNA and miRNA interactions in the tumorigenesis and metastasis of several cancers, like breast and colorectal cancer [17–20], underscoring their relevance in HCC. Specific circRNAs, such as circMTO1 [21] and circGPR137B [22], have been found to influence HCC by acting as sponges. circMTO1 suppresses HCC progression by acting as the sponge of oncogenic miR-9 to promote p21 expression [21]. circGPR137B inhibits HCC tumorigenesis and metastasis through the circGPR137B/miR-4739/FTO feedback loop [22]. Newer research shows that circZFR acts as a competitive endogenous RNA by sponging microRNA-624-3p to regulate WEE1 expression, thereby promoting HCC proliferation, migration, and invasion [13]. This growing evidence underscores the integral role of circRNAs in HCC development.

Recent investigations into circRNAs, particularly circ_0000069, have shown its upregulation in various cancers. For instance, in cervical cancer, circ_0000069 promotes cell proliferation, motility, and invasion by acting as a miRNA sponge and elevating CPEB4 levels [23]. In renal cell carcinoma, circ_0000069 contributes to cancer progression and glutamine metabolism by sequestering miR-125a-5p and upregulating SLC1A5 expression, with its knockdown resulting in reduced tumor growth [24]. High levels of circ_0000069 in pancreatic cancer correlate with poorer survival, and its reduction hampers tumor growth, suggesting its potential as a therapeutic target [25]. This body of research highlights the significant role of circ_0000069 in cancer biology and suggests that targeting this molecule may open new therapeutic avenues. However, the role of circ_0000069 in the progression of HCC is still not well understood, meriting further investigation into its functional importance.

In our study, we identified differentially expressed circRNAs, mRNAs, and miRNAs between HCC and adjacent normal tissues from available datasets to construct a competitive endogenous RNA (ceRNA) network. Our in vitro and in vivo experiments subsequently validated that circ_0000069 is significantly upregulated in HCC tissues and plays a functional role. These findings enhance our understanding of circ_0000069’s potential role in HCC progression.

## Materials and methods

### Data source and preprocessing

The raw data were selected and downloaded from the public database Gene Expression Omnibus (GEO, https://www.ncbi.nlm.nih.gov/geo/). CircRNA profiling datasets (accession GSE97332) from HCC tumor tissues (*n* = 7) and corresponding normal tissues (*n* = 7) were obtained using the GPL19978 platform (Agilent-069978 Arraystar Human CircRNA microarray V1). To mitigate the impact of formalin storage on sample quality, mRNA and miRNA samples preserved in formalin from the TCGA dataset were excluded. The remaining samples were used to generate an expression matrix for subsequent analyses.

### Bioinformatics analysis

Genes demonstrating a significant mean difference (ANOVA, *P* < 0.05) across all samples were subjected to principal component analysis (PCA). The correlation coefficient for gene expression values between the samples was calculated and visualized as a heatmap. Differential expression values were computed using the R packages Limma [26] (for chip data) and edgeR (for sequencing data) [27]. Differentially expressed genes (DEGs) were identified with a fold change (FC) greater thand 2 and a P value less than 0.05. Tools such as miRanda, miRDB, miTarBase, TargetScan, miRMap, and StarBase were employed to predict binding relationship between miRNAs, mRNAs and circRNAs. The circRNA-miRNA-mRNA network was constructed using Cytoscape V3.7. Fisher's exact test was utilized for enrichment analyses to identify the enriched Gene Ontology (GO) terms and Kyoto Encyclopedia of Genes and Genomes (KEGG) pathways associated with genes closely related to a specific group of genes.

### Patients and clinical specimens

Tumor tissues and adjacent non-tumor tissues of 30 paired HCC cases were collected from at Hunan Cancer Hospital between February 2022 and December 2022. The study was approved by the Ethics Committee of Hunan Cancer Hospital (Approval No. 2023–092). Signed informed consent was obtained from each patient. The inclusion criteria were as follows: patients diagnosed with HCC, comprehensive clinical data availability, absence of distant metastasis, no prior systemic therapy, radiotherapy, or chemotherapy, HBsAg positivity, and age over 18 years. Exclusion criteria were cardiovascular and cerebrovascular diseases, other malignancies, and pregnancy or lactation. Fresh tissues were immediately frozen and stored in liquid nitrogen prior to RNA extraction. All samples underwent laboratory pathological examination to verify the diagnosis.

### Real-time quantitative PCR

A real-time quantitative PCR (RT-qPCR) assay was performed according to our previous study [28]. Total RNA was extracted and quantified from 30 pairs of normal/tumor tissue samples and cell samples from each group. cDNA was synthesized from the RNA using reverse transcription. RT-qPCR was performed with these primers to quantitatively assess circRNA levels. Analyzed the results using the 2^−ΔΔCt^ method. Relative expression of circRNA was normalized to human 18S rRNA. Relative expression levels of specific genes were normalized to the reference gene GAPDH. The PCR primers used in this study are listed in Table 1.

**Table 1:** RT-qPCR Primer sequences in this study

Gene	Primer	
circ_0000069	forward	5′-CTACTTCAGGCACAGGTCTTC-3′
	reverse	5′-CTGACTCACTGGATGAGGACT-3′
CCL25	forward	5′-GCCTGCTGCGATATTCTAC-3′
	reverse	5′-GCTGATGGGATTGCTAAAC-3′
MAP2K1	forward	5′-CAATGGCGGTGTGGTGTTC-3′
	reverse	5′-GATTGCGGGTTTGATCTCCAG-3′
18S rRNA	forward	5′-GGGCCACTTGGCATACA-3′
	reverse	5′-CGAACCTCCGACTTTCGTTCT-3′
GAPDH	forward	5′-GGGAGCCAAAAGGGTCAT-3′
	reverse	5′-GAGTCCTTCCACGATACCAA-3′

### Western blotting

Western blotting assay was performed according to our previous study [28]. Cells and tissue samples were homogenized and lysed to extract proteins. Proteins were separated based on molecular weight by SDS-PAGE and subsequently transferred from the gel to a PVDF membrane. The membrane was blocked to prevent non-specific binding and incubated with a primary antibody specific to the target protein. After washing, a labeled secondary antibody was applied, which binds to the primary antibody. The target protein was detected using a chemiluminescent method after further washes, providing both qualitative and quantitative insights into protein levels. For western blotting, the primary antibodies were MAP2K1/MEK1 antibody (#NBP2-67358, Novus Biologicals) and CCL25/TECK antibody (#MAB3341, Novus Biologicals). Prior to hybridization with antibodies, the membranes were cut to isolate specific regions corresponding to the target proteins. This was done to ensure precise detection and facilitate the subsequent analysis of the specific bands of interest.

### In vitro experiments

The non-invasive human liver cell line of LO2 (normal), the low invasive human HCC cell line of Hep3B, and the highly invasive human HCC cell lines of SNU-387, HepG2, Huh7, SMMC-7721, and HCCLM3 were obtained from American Type Culture Collection (ATCC, Manassas, VA, USA). They were cultured in DMEM supplemented with 20% fetal bovine serum (FBS), 2 mM L-glutamine, 100 units/mL of penicillin, and 100 µg/mL of streptomycin (complete medium, Gibco, Auckland, New Zealand) at 37 °C in a 5% CO2 incubator. Cellular migratory and invasive capabilities were assessed using wound healing and transwell invasion assays, as outlined in established protocols [29, 30].

### Animal experiments

C57BL/6 nude mice were obtained from Hunan Slack Jingda Experimental Animal (Changsha, China, the experimental animal production license number: SCXK (Xiang) 2016–0002). The mice were injected with 4 × 10^6^ WT Huh7 and HepG2 cells (control), circ_0000069-knockdown Huh7 and HepG2 cells, or circ_0000069-knockdown and the CCL25-overexpression Huh7 and HepG2 cells (15 nude mice were randomly divided into 3 groups, 5 in each group). All animal experiments adhered to the ARRIVE guidelines and were conducted following the National Institutes of Health Guide for the Care and Use of Laboratory Animals (NIH Publication No. 8023, revised 1978). The progression of HCC tumor transplants was monitored by measuring their volume at five-day intervals for a duration of 30 days post-inoculation. Upon completion of the study, CO2 euthanasia was performed, and the xenografts were documented photographically for subsequent analysis. All animal experiment protocols received approval from the Animal Ethics Committee of Guangxi Medical University.

### Statistical analysis

Data are expressed as an average ± standard error of the mean (SEM). Differences between groups were analyzed using one-way ANOVA followed by Tukey's post hoc test. A two-tailed t-test was applied to evaluate inter-group disparities using SPSS 10.0 for Windows. A *P*-value less than 0.05 was considered statistically significant (*p < 0.05, **p < 0.01, ***p < 0.001).

## Results

### Identification of differentially expressed mRNAs, miRNAs and circRNAs

We collected 371 HCC tumor samples and 50 normal liver samples from the TCGA-LIHC database to perform differential expression analysis. Analysis revealed 612 genes up-regulated and 1173 genes down-regulated in HCC tumor samples compared to the normal liver samples (Table S1).

**Fig. 1:**
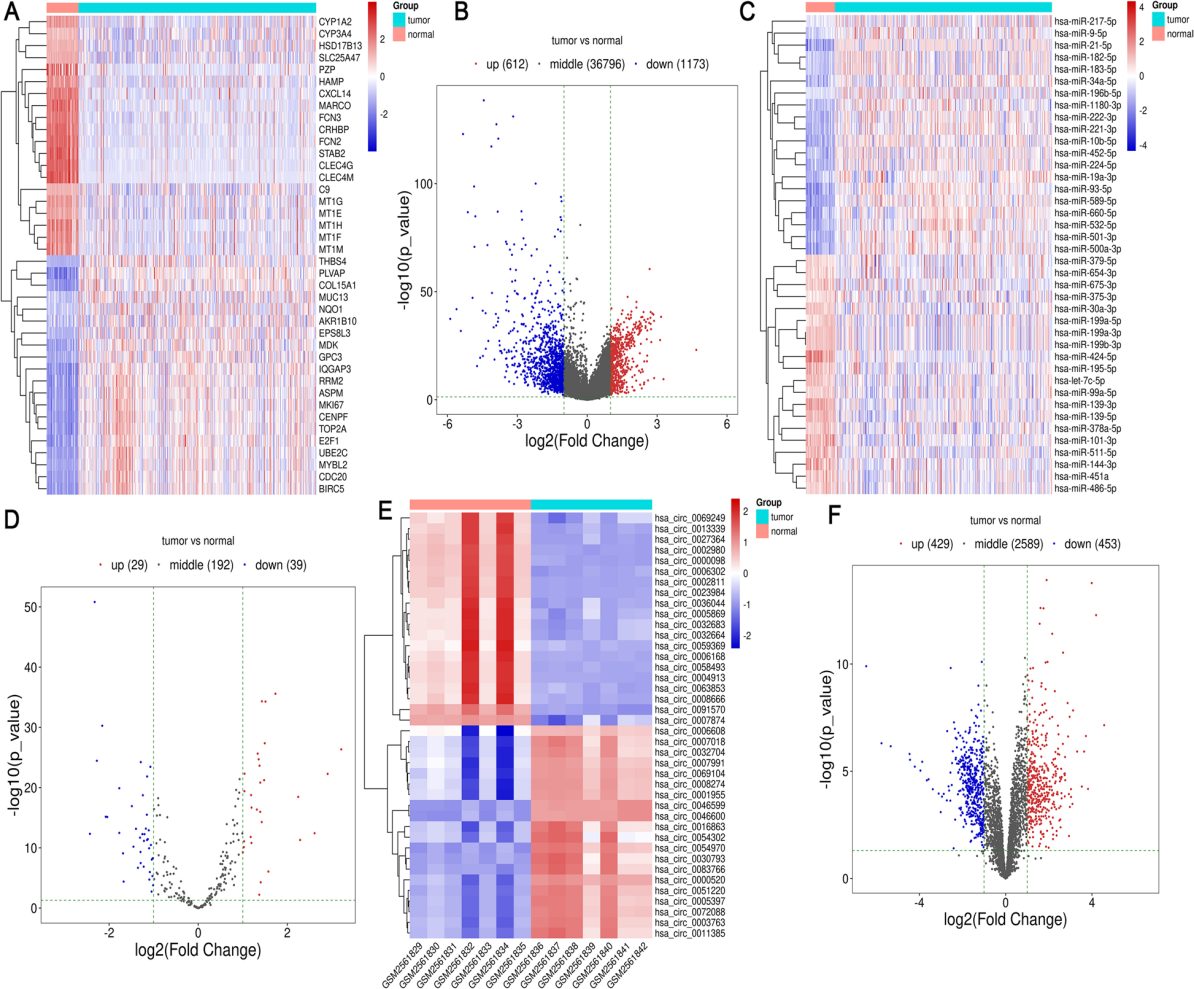
Differential analysis of HCC samples and normal samples. **A** Heatmap of DEGs identified in the analysis. The genes are ranked in descending order of log_2_FC between the HCC and normal samples. The top 20 DEGs are shown in the heatmap, with the most upregulated genes in red and the most downregulated genes in blue. **B **Volcano plot of DEGs. Each dot on the plot represents a gene, with the x-axis representing the log_2_FC and the y-axis representing the -log10 *p*-value. The red dots represent genes that are significantly upregulated in HCC samples, while the blue dots represent genes that are significantly downregulated. **C **Heatmap of differentially expressed miRNAs, with the top 20 miRNAs sorted in descending order of log_2_FC. Like the gene heatmap, the miRNA heatmap uses a color scheme to indicate the upregulation or downregulation of miRNAs in HCC samples compared to normal samples. **D** Volcano plot of miRNAs. **E **Heatmap of differentially expressed circRNAs (first twenty circRNAs sorted in descending order of log_2_FC). **F** Volcano plot of differentially expressed circRNAs

We then produced a heatmap to illustrate the first twenty differentially expressed genes, ranked by log2FC (Fig. 1A). The heatmap disclosed two principal clusters: one comprising genes up-regulated in HCC samples yet down-regulated in normal tissues, and the other with genes up-regulated in normal tissues but down-regulated in HCC samples. A volcano plot succinctly illustrated the differentially expressed genes based on their fold change (FC) and *P*-value (presented in Fig. 1B).

For miRNA analysis, we extracted 364 HCC miRNA-seq samples and 49 normal miRNA-seq samples from the TCGA-LIHC database. The results identified 29 up-regulated and 39 down-regulated miRNAs, hinting at their possible involvement in HCC progression (Table S2). A subsequent heatmap of the top twenty differentially expressed miRNAs, organized by descending log2FC, showed two distinct clusters (Fig. 1C). A volcano plot was also employed to showcase the distribution of the differentially expressed miRNAs, taking into account their FC and statistical significance (Fig. 1D).

In the circRNA analysis, we sourced seven HCC circRNA-seq samples and seven normal circRNA-seq samples from the GSE97332 dataset. This led to the identification of 429 up-regulated and 453 down-regulated circRNAs compared to normal samples (illustrated in Fig. 1E). The differential expression of circRNAs was analyzed using edgeR. Furthermore, a volcano plot was applied to visualize the distribution of differentially expressed circRNAs based on the FC and associated statistical significance (*P*-value) (depicted in Fig. 1F). Notable up-regulated circRNAs in HCC samples included hsa_circ_0072088, hsa_circ_0046600, hsa_circ_00053979, and hsa_circ_0000069, among others (detailed in Table S3).

### Construction of the ceRNA network

**Fig. 2:**
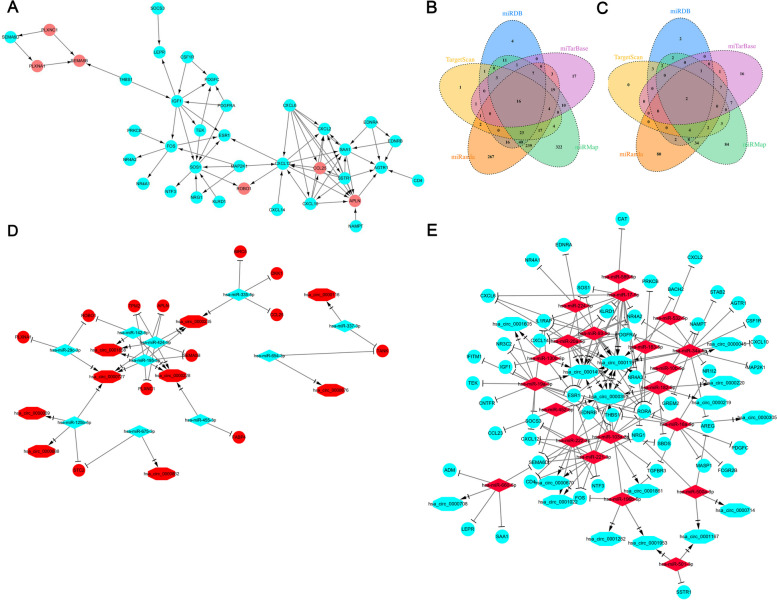
CeRNA network analysis. **A **The network between CCL25 and MAP2K1. **B **Venn diagram of up-regulated miRNA_down-regulated mRNA pairs identified in five different databases including miRanda, miRDB, miTarBase, TargetScan, miRMap, showing the overlap pairs between the five different databases. **C **The venn diagram of down-regulated miRNA_up-regulated mRNA pairs identified in five different databases. **D **The network of up-regulated mRNA, down regulated miRNAs, and up-regulated circRNAs. **E **The network of down-regulated mRNAs, up regulated miRNAs, and down-regulated circRNAs. The circles indicate genes, the rhombus represents miRNAs and the octagon represents circRNAs. Cool color means down regulation, and the degree of down regulation is weakened with the color from deep to light. Warm color means up regulation. This network provides the regulatory interactions among these RNAs in HCC

Additionally, we searched the differentially expressed mRNAs related to immunity in the ImmPort database. Finally, 199 up-regulated immune-related genes, including KLRD1, MAP2K1, FCGR3A, and other mRNAs (Table S4), and 41 down-regulated immune-related genes were detected, respectively. We first established a PPI network, and through analysis, we identified a potential regulatory relationship between CCL25 and MAP2K1 (Fig. 2A). Using public databases, we established the miRNA-mRNA interactions between the aforementioned differentially expressed mRNAs and miRNAs (Fig. 2B, C), ensuring at least three interaction pairs were confirmed by the mentioned databases. Specifically, we identified 131 pairs of up-regulated miRNAs with down-regulated genes (detailed in Table S5) and 26 pairs of down-regulated miRNAs with up-regulated genes (found in Table S6). Furthermore, from the StarBase database, we extracted 60 pairs of up-regulated miRNAs with down-regulated circRNAs (Table S7) and 40 pairs of down-regulated miRNAs with up-regulated circRNAs (Table S8). For instance, circRNA_0000069 was shown to connect to four distinct miRNAs within the ceRNA network. Based on the aforementioned miRNA-mRNA and miRNA-circRNA pairs (Table S7 and Table S8), we constructed the circRNA-related ceRNA regulatory networks using Cytoscape (illustrated in Fig. 2D and E).

### Expression analysis of circ_0000069 in HCC cells and clinical samples

**Fig. 3:**
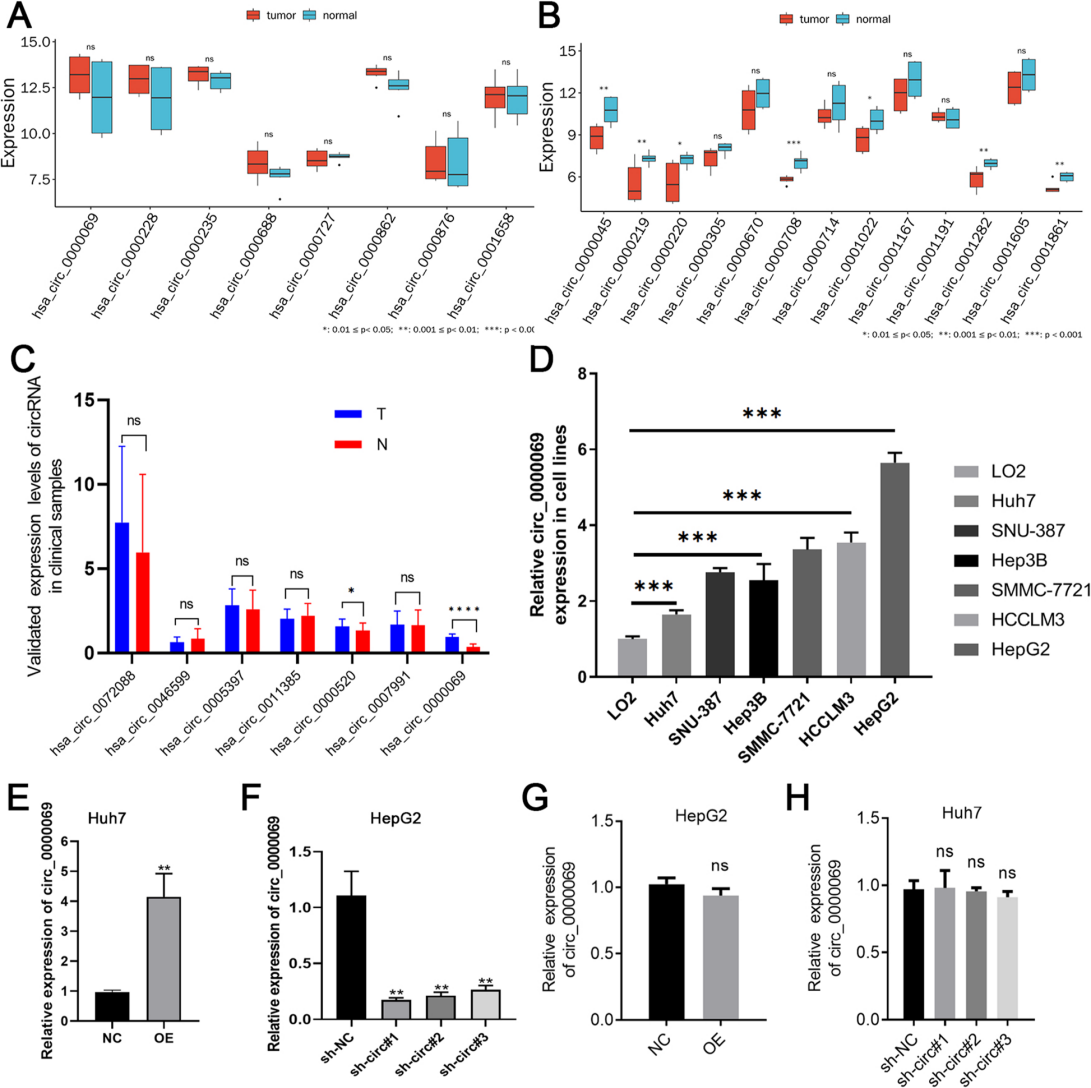
The role of circ_0000069 expression in HCC cells. **A** Up-regulated circRNAs in the GSE164803 dataset. **B **Down-regulated circRNAs in the GSE164803 dataset. **C** The relative expression levels of hsa_circ_0072088, hsa_circ_0046599, hsa_circ_0005397, hsa_circ_0011385, hsa_circ_0000520, hsa_circ_0007991, and hsa_circ_0000069 in the control group (normal tissue, N) and the treatment group (tumor tissue, T). **D **The relative expression of circ_0000069 in different HCC cell lines (SNU-387, HepG2, Hep3B, Huh7, SMMC-7721, and HCCLM3) and a normal epithelial cell line (LO2). **E **The relative expression level of circ_0000069 in normal condition (NC) and the circ_0000069- overexpression condition (OE) in Huh7 cell lines. **F **The relative expression level of circ_0000069 in normal condition (sh-NC) and the circ_0000069-silenced condition (sh-circ0000069) in HepG2 cells. **G **The relative expression level of circ_0000069 in normal condition (NC) and the overexpression circ_0000069 condition (OE) in HepG2 cell lines. **H **The relative expression level of circ_0000069 in the normal condition (sh-NC) and the circ_0000069-silenced condition (sh-circ0000069) in Huh7 cell lines

To confirm the validity and reliability of our ceRNA network, we detected the expression of selected differential circRNAs in clinical samples. We first analyzed the up-regulation and down-regulation of circRNAs in the normal control group and the tumor tissue group according to the data set GSE164803 (Fig. 3A and B). Subsequently, we began by validating the expression of seven circRNAs in HCC and adjacent non-tumorous tissue samples. Notably, circ_0000069 showed the most significant up-regulation in tumor samples compared to adjacent controls (Fig. 3C).

Furthermore, the expression levels of circ_0000069 were quantified using qPCR in various cell lines: SNU-387, HepG2, Hep3B, Huh7, SMMC-7721, HCCLM3, and LO2 normal epithelial cells. The results indicated that circ_0000069 was most abundant in HepG2 cells and least expressed in Huh7 cells (Fig. 3D). After transfecting and culturing Huh7 and HepG2 cells for forty-eight hours, we observed an increase in circ_0000069 expression in the Huh7 overexpression group (Fig. 3E), while no significant change was detected in HepG2 cells (Fig. 3G). In contrast, silencing circ_0000069 decreased its expression in HepG2 cells (depicted in Fig. 3F) , while it did not significantly affect expression in Huh7 cells (Fig. 3H). Consequently, for the overexpression studies, Huh7 cells were selected, while HepG2 cells were used for silencing experiments in further investigations.

### The effects of circ_0000069 on CCL25 and MAP2K1 expression, biological behavior of HCC cells

**Fig. 4:**
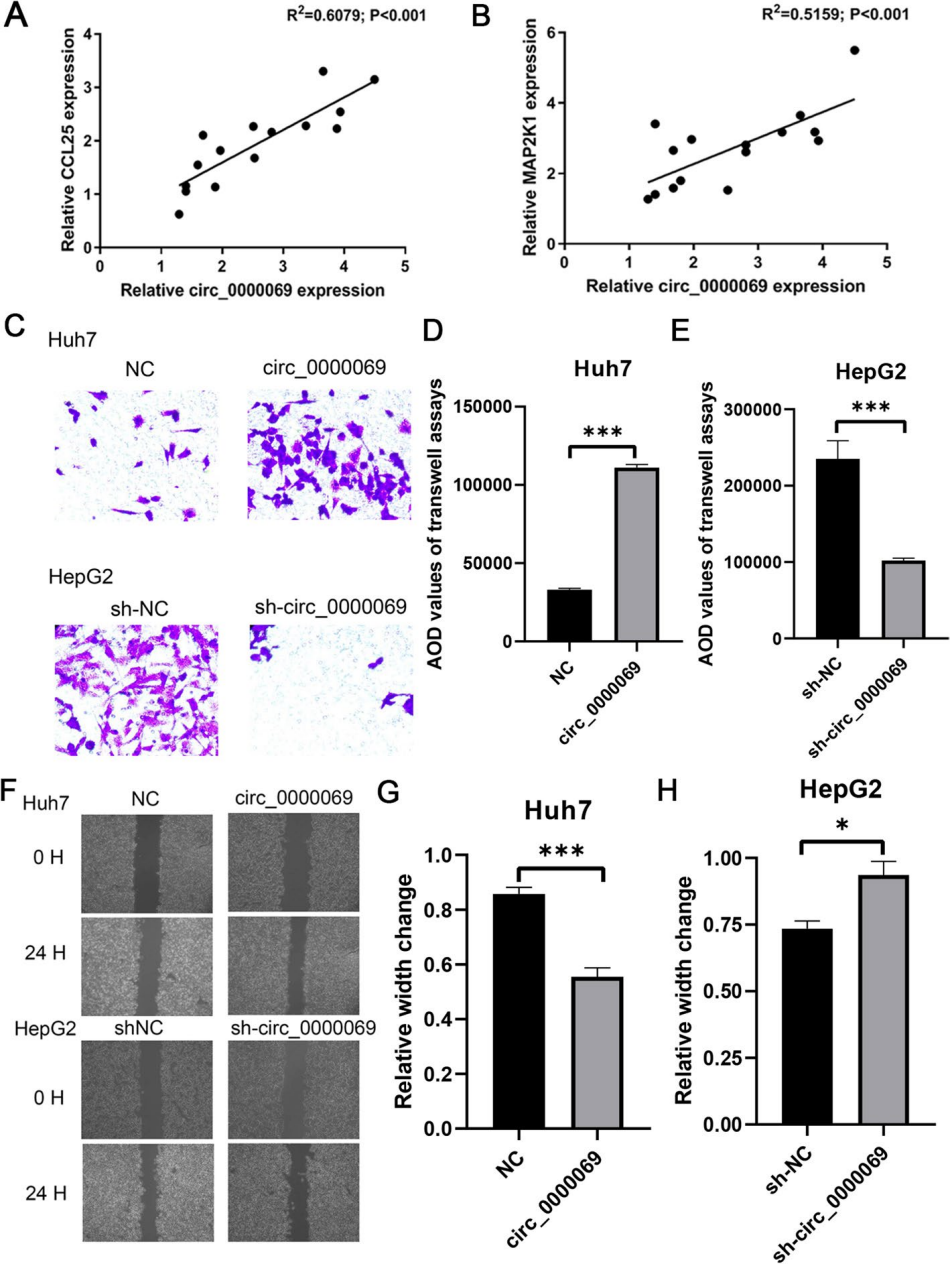
Aberrant expression of circ_0000069 and its impact on the hyperplasia, metastatic potential, and invasiveness of HCC cells. **A-B** The correlation between the expression of circ_0000069 and MAP2K1 (A) and CCL25 (B). **C **Transwell assays of normal condition (NC), and the overexpression circ_0000069 (circ_0000069) of Huh7 cell lines, and normal condition (sh-NC), and the silencing circ_0000069 (sh-circ_0000069) of HepG2 cell lines. **D-E** Quantification from C in Huh7 and HepG2 cell lines, respectively. The two-independent t-test was used to analyze the differences in the AOD values of the transwell assays. **F **Wound-healing assays of the normal condition (NC), and the overexpression circ_0000069 (circ_0000069) of Huh7 cell lines, and normal condition (sh-NC), and the silencing circ_0000069 (sh-circ_0000069) of HepG2 cell lines. **G **The relative width of the overexpression circ_0000069 (circ_0000069) of Huh7 cell lines compared with normal condition (NC) in F. **H** The relative width of the silencing circ_0000069 (sh-circ_0000069) of HepG2 cell lines compared with normal condition (sh-NC) in F

In order to elucidate the regulatory mechanism of circ_0000069 with the hub genes, we examined the expression of circ_0000069, CCL25, and MAP2K1 in tumor tissues and adjacent non-tumor tissues from 30 paired HCC patients. A correlation analysis was conducted between circ_0000069 and the hub genes. Pearson correlation analysis showed a significant positive correlation between circ_0000069 and CCL25 (R = 0.6079, *P* < 0.001) (Fig. 4A), as well as between circ_0000069 and MAP2K1 (R = 0.5159, *P* < 0.001) (Fig. 4B).

Consequently, the impact of circ_0000069 on biological behavior of HCC cells, including the expression of MAP2K1 and CCL25, was further investigated. To investigate the effect of circ_0000069 expression on HCC cell invasiveness, we performed transwell experiments. We assessed the invasive capacity by measuring AOD values. The results showed that overexpression of circ_0000069 in Huh7 cells led to an increase in AOD values, with the control group at 33,071 ± 1,231 and the overexpression group at 111,071 ± 3,861. However, upon knockdown of circ_0000069, the AOD values decreased from 235,203 ± 19,406 to 102,214 ± 2,273 (Fig. 4C-E). Furthermore,wound healing was used to measure the migration ability of HCC cells affected by abnormal expression of circ_0000069. After gently scratching Huh7 and HepG2 cells with a sterile 200 µL pipette tip at 0 and 24 h, the wound width was measured and the relative wound width was presented. Our analysis revealed that in Huh7 cells, overexpression of circ_0000069 resulted in a decrease in cell width from 0.8568 ± 0.0347 μm at 0 h to 0.5547 ± 0.0465 μm after 24 h of culture. However, in HepG2 cells, knockdown of circ_0000069 led to an increase in cell width from 0.7344 ± 0.0420 μm at 0 h to 0.9359 ± 0.1019 μm after 24 h (Fig. 4F-H).

**Fig. 5:**
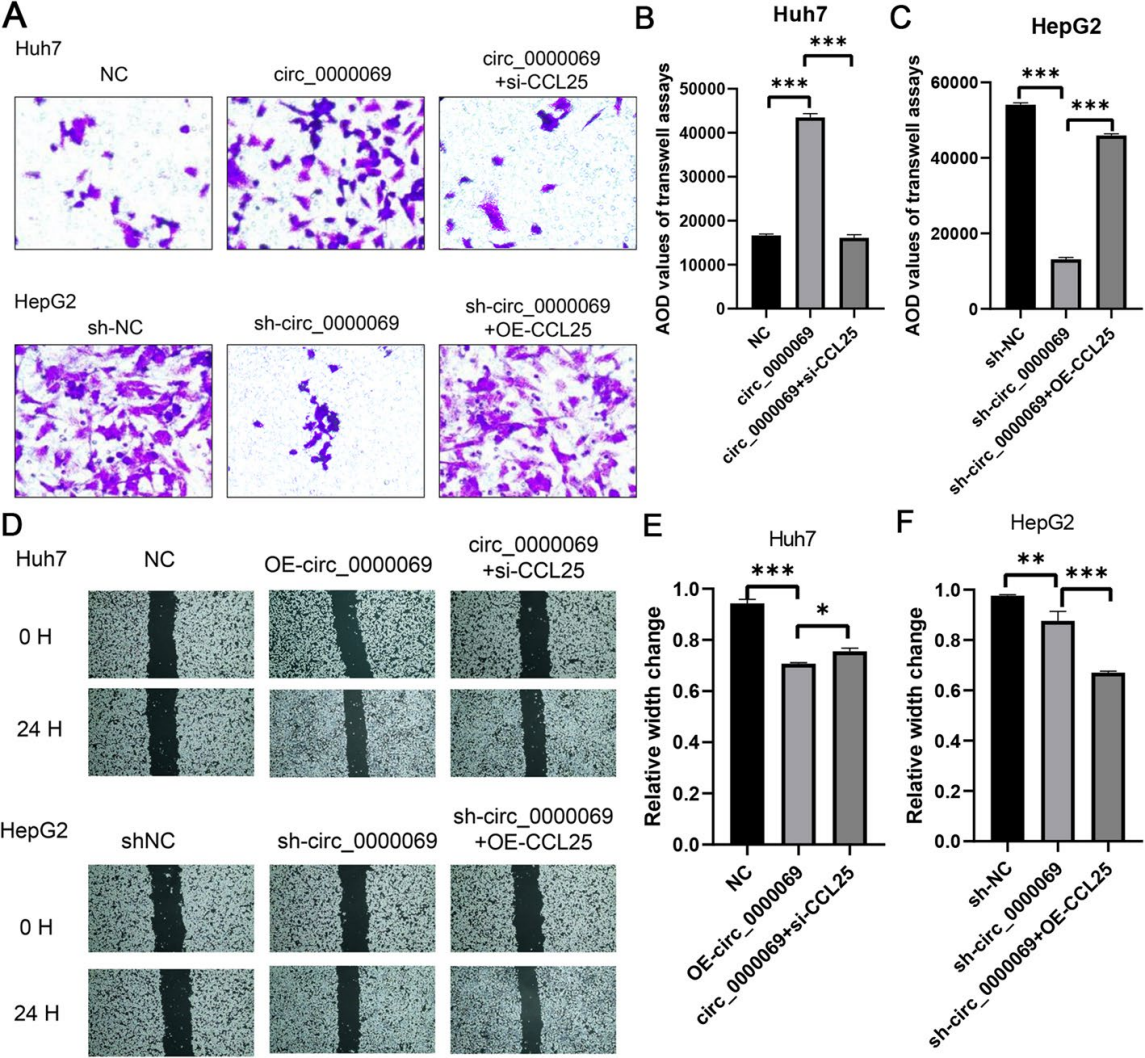
Aberrant expression of CCL25 on hyperplasia, metastatic ability, and invasiveness of HCC cells through the expression of circ_0000069. **A** The transwell assays of normal condition (NC), the overexpression circ_0000069 (circ_0000069), and the overexpression circ_0000069 and the silencing CCL25 (circ_0000069 + si-CCL25) of Huh7 cell lines, and normal condition (sh-NC), the silencing circ_0000069 (sh-circ_0000069), and the silencing circ_0000069 and the overexpression CCL25 (sh-circ_0000069 + OE-CCL25) of HepG2 cell lines. **B-C** Quantification from A in Huh7 and HepG2 cell lines, respectively. The two-independent t-test analyzed the difference of transwell assays' AOD values. **D** Wound-healing assays of normal condition (NC), the overexpression circ_0000069 (OE-circ_0000069), and circ_0000069 + si-CCL25 of Huh7 cell lines, and normal condition (sh-NC), the silencing circ_0000069 (sh-circ_0000069), and sh-circ_0000069 + OE-CCL25 of HepG2 cell lines. **E** The relative width of circ_0000069 (OE-circ_0000069), and circ_0000069 + si-CCL25 of Huh7 cell lines compared with normal condition (NC) in D. **F** The relative width of the silencing circ_0000069 (sh-circ_0000069), and sh-circ_0000069 + OE-CCL25 of HepG2 cell lines compared with normal condition (sh-NC) in D

Lentiviral vectors were constructed for both silencing (circ_0000069 + si-CCL25) and overexpression (sh-circ_0000069 + OE-CCL25) of CCL25. Subsequently, a transwell assay was conducted to study the effect of CCL25 on the invasiveness of HCC cells. Overexpression of circ_0000069 significantly promoted the invasiveness of Huh7 cells, while silencing CCL25 following the overexpression of circ_0000069 reduced the AOD value from 43,489 ± 919 to 16,118 ± 558 (Fig. 5A, B). Additionally, knocking down circ_0000069 significantly inhibited the invasiveness of HepG2 cells, and overexpressing CCL25 after knocking down circ_0000069 increased the AOD value from 13,112 ± 504 to 45,921 ± 356 (Fig. 5A, C), indicating a competitive relationship between CCL25 and circ_0000069.

Subsequently, we conducted a wound healing assay to investigate the effect of CCL25 on the migratory capacity of HCC cells. Our analysis showed that the cell width was 0.7068 ± 0.0058 in the circ_0000069 overexpression group. When CCL25 was silenced following the overexpression of circ_0000069, the measured cell width increased to 0.756 ± 0.017 (Fig. 5D, E). In the circ_0000069 knockdown group, the cell width was 0.8765 ± 0.0527. Upon overexpression of CCL25 in the circ_0000069 knockdown background, the cell width decreased to 0.6705 ± 0.0085 (Fig. 5D, F). These findings suggest that CCL25 acts as a negative regulator of HCC cell hyperplasia and invasiveness, counteracting the oncogenic effect of circ_0000069. The functional crosstalk between CCL25 and circ_0000069 highlights the complexity of the regulatory network in HCC pathogenesis and provides potential therapeutic targets for HCC treatment.

### Circ_0000069 promotes HCC angiogenesis by the upregulation of CCL25

**Fig. 6:**
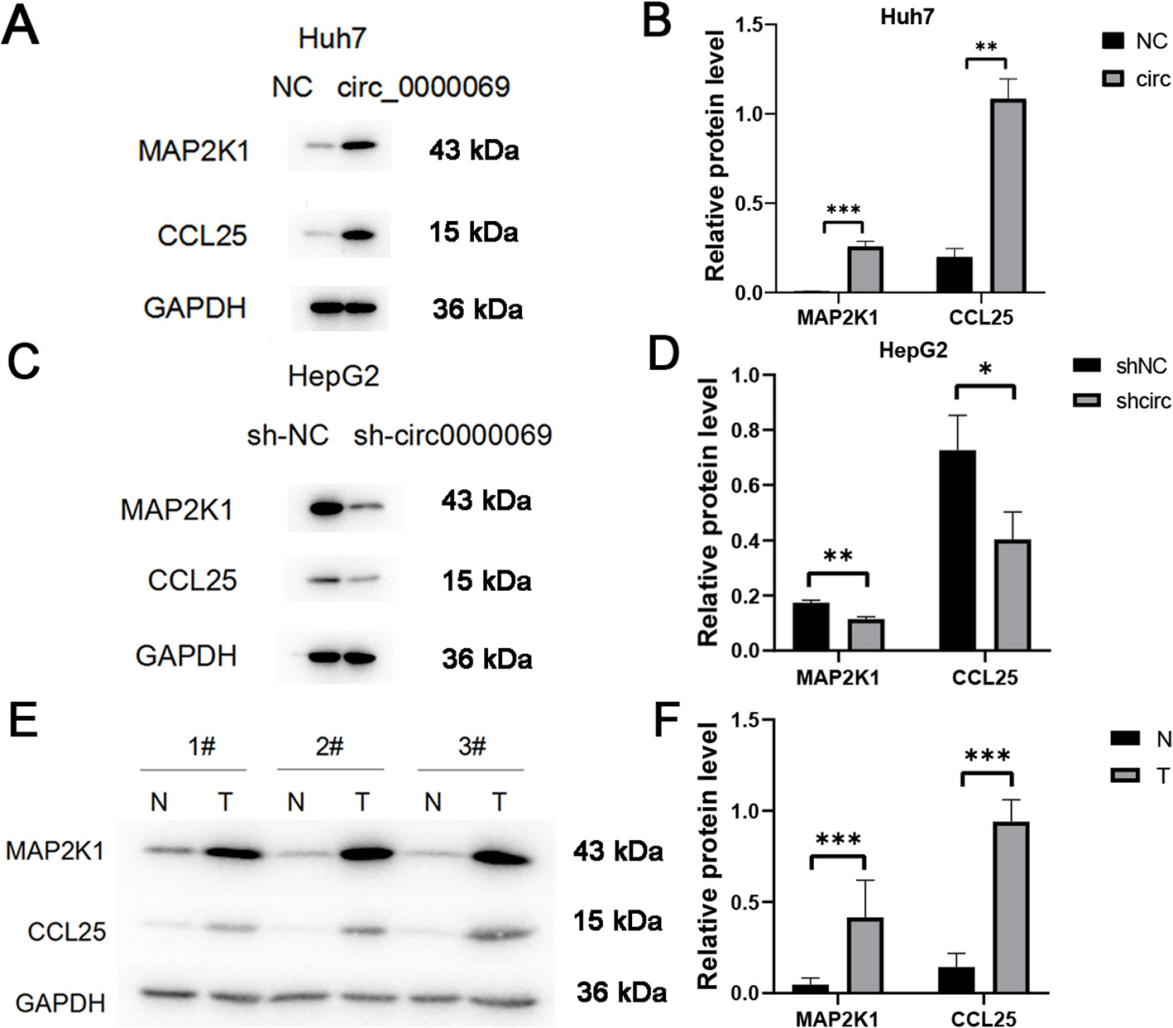
Validation of the role of circ_0000069 in MAP2K1 and CCL25 expression. **A** The protein levels of MAP2K1, CCL25 and control GAPDH in the control group (NC) and the circ_0000069- overexpression group (circ_0000069) of Huh7 cell lines were determined by Western blot (WB) analysis. **B** The relative protein levels of MAP2K1 and CCL25 in the control group (NC) and the circ_0000069-overexpression group (circ_0000069) of Huh7 cell lines from A.** C** The abundances of protein MAP2K1, CCL25 and control GAPDH in the control group (sh-NC) and the circ_0000069-silenced group (sh-circ_0000069) of HepG2 cell lines were determined by WB analysis. **D** The relative protein levels of MAP2K1 and CCL25 in the control group (sh-NC) and the circ_0000069-silenced group (sh-circ_0000069) of HepG2 cell lines from B. **E **The abundances of protein MAP2K1, CCL25 and control GAPDH in the normal tissues (N) and the tumor tissues (T) of three patients were determined by WB analysis. **F** The relative protein levels of MAP2K1 and CCL25 in the normal tissues (N) and the tumor tissues (T) from E

To determine the impact of circ_0000069 on the expression of MAP2K1 and CCL25 in Huh7 and HepG2 cell lines, we performed western blot assays. Overexpression of circ_0000069 in Huh7 cell lines resulted in increased levels of MAP2K1 and CCL25 compared to the control groups (Fig. 6A and B). On the other hand, knockdown of circ_0000069 in HepG2 cell lines resulted in reduced levels of these proteins relative to controls (Fig. 6C and D). Additionally, MAP2K1 and CCL25 expression was significantly elevated in HCC tissues in comparison to adjacent tissues (Fig. 6E and F). These findings bolster the proposed regulatory function of circ_0000069 in the progression of HCC.

**Fig. 7:**
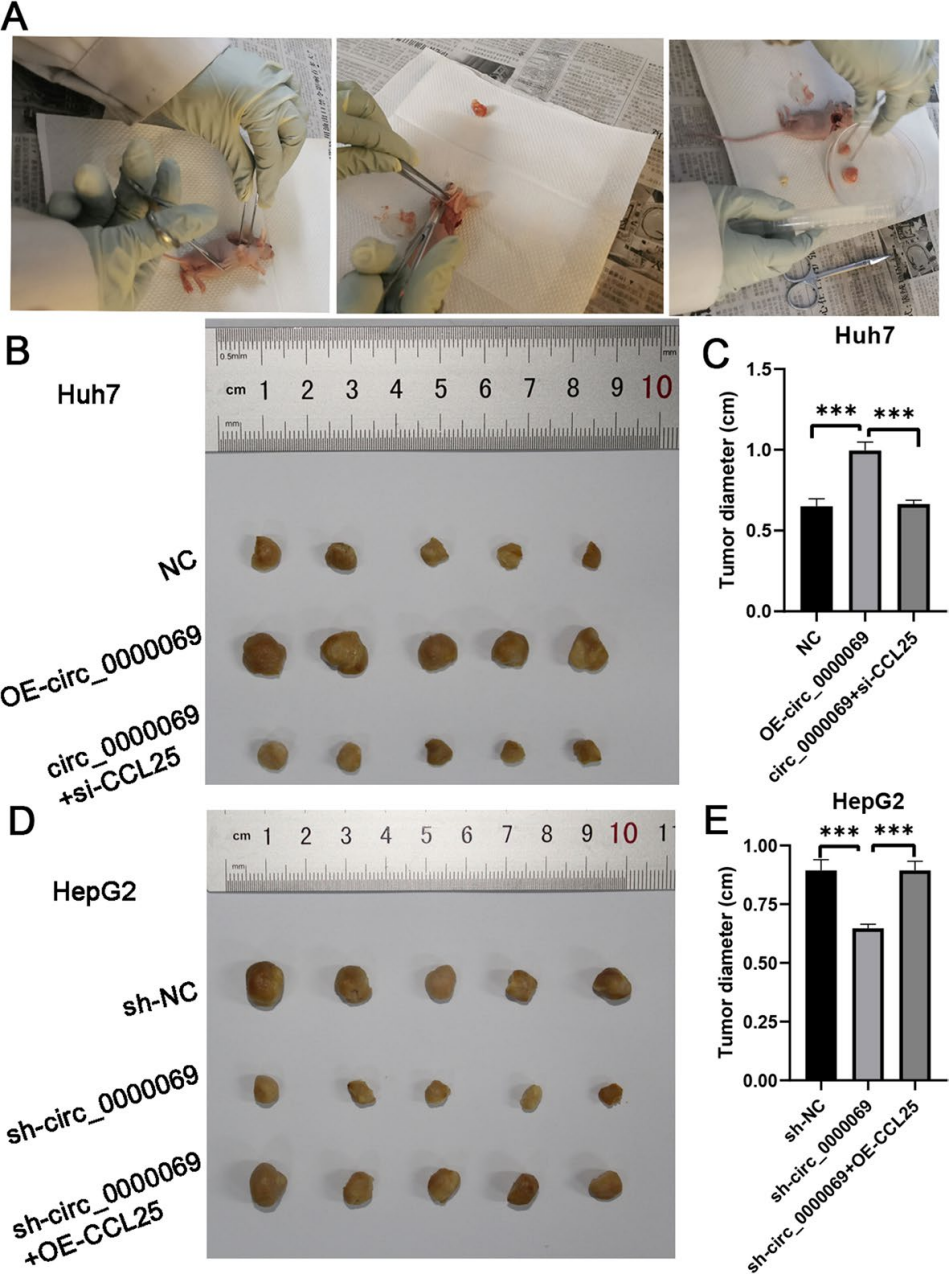
Overexpression of circ_0000069 promotes tumor growth in vivo. **A** Mice of in vivo xenograft models. **B** The tumorigenesis experiment of normal condition (NC), the overexpression circ_0000069 (OE-circ_0000069), and the overexpression circ_0000069 and the silencing CCL25 (circ_0000069 + si-CCL25) of Huh7 cell lines. **C** Tumor diameter of normal condition (NC), the overexpression circ_0000069 (OE-circ_0000069), and the overexpression circ_0000069 and the silencing CCL25 (circ_0000069 + si-CCL25) of Huh7 cell lines from B.** D** The tumorigenesis experiment of normal condition (sh-NC), the silencing circ_0000069 (sh-circ_0000069), and the silencing circ_0000069 and the overexpression CCL25 (sh-circ_0000069 + OE-CCL25) of HepG2 cell lines. **E** Tumor diameter of normal condition (sh-NC), the silencing circ_0000069 (sh-circ_0000069), and the silencing circ_0000069 and the overexpression CCL25 (sh-circ_0000069 + OE-CCL25) of HepG2 cell lines from D

To investigate the combined effects of circ_0000069 and CCL25 on tumor growth in vivo, we carried out subcutaneous injection experiments. Cells with silenced circ_0000069 and those with overexpressed circ_0000069, both also altering CCL25 expression, were injected into the armpits of nude mice (Fig. 7A). Compared to the control group, overexpression of circ_0000069 notably promoted the growth of implanted Huh7 tumors, an effect that was mitigated by silencing CCL25 (Fig. 7B and C). In contrast, silencing circ_0000069 led to a decrease in the growth of implanted HepG2 tumors, an effect that was reversed by overexpressing CCL25 (Fig. 7D and E). These findings corroborate that circ_0000069 overexpression fosters xenograft growth in vivo, and that this pro-tumorigenic effect can be negated by downregulating CCL25.

## Discussion

HCC is among the deadliest cancers, frequently associated with aberrant expression of circRNAs [31]. However, the precise roles of many circRNAs are still enigmatic. To elucidate the complex regulatory network underlying HCC, we identified a spectrum of differentially expressed mRNAs, miRNAs, and circRNAs, and constructed a corresponding ceRNA network. Within this framework, we discovered a novel circRNA, circ_0000069, which appears to play a facilitative role in HCC tumorigenesis. Our subsequent functional and mechanistic studies indicate that circ_0000069 enhances the invasive and migratory behaviors of HCC cells, signifying its likely contribution to the cancer's progression.

The potential of bioinformatics to demystify the molecular intricacies of HCC has been underscored in recent literature, opening new pathways for both diagnostic and therapeutic interventions [32]. The integration of phenotypic data with comprehensive molecular profiling allows for the unravelling of disease mechanisms and the pinpointing of new therapeutic targets. The investigation into non-coding RNAs—including circRNAs and miRNAs—and their interplay with mRNAs, is gaining momentum as a promising research avenue [33, 34]. Several studies have reported on circRNAs, such as circMRPS35 [35], circMDK [36], and circVAMP3 [37], which are up-regulated in HCC and have been implicated promoting tumorigenesis. These molecules are involved in critical regulatory processes and may serve as biomarkers for early detection or as targets for precision medicine strategies in HCC [16].

In our study, we identified a significant upregulation of circ_0000069 in HCC samples, which suggests its potential role as an oncogenic molecule. Through an integrated approach, we analyzed the circRNA-miRNA-mRNA network within HCC. Following this, we specifically validated the expression levels of seven circRNAs in both HCC and adjacent non-cancerous tissue samples. Our results showed a significant upregulation of circ_0000069 in HCC cell lines and tissue samples. Previous research has linked the dysregulation of circ_0000069 to cervical squamous cell carcinoma [38] and an overexpression in colorectal cancer [39]. The oncogenic potential of circ_0000069 is becoming increasingly recognized, particularly in cervical cancer [40, 41]. In our study, overexpression of circ_0000069 was associated with increased cell proliferation, metastatic capability, and invasiveness in HCC models, both in vitro and in vivo. This aligns with findings by Ye et al., who observed that lowering circ_0000069 levels reduced these malignant properties in pancreatic cancer cells and decreased the growth of pancreatic cancer xenograft tumors in vivo [25]. Overexpression of hsa_circ_0000069 in HCC may disrupt the normal regulatory cascade involving miRNAs and their mRNA targets, leading to cellular dysfunctions that favor cancer progression. Our data support a growing body of literature implicating circRNAs in the proliferation, migration, and invasiveness of cancer cells [23–25, 40, 41].

CCL25 is a chemokine implicated in immune responses and tumor biology. It acts as the ligand for CCR9, enhancing the CCR9/CCL25 signaling pathway, which has been shown to modulate cancer cell migration, invasion, and drug resistance [42]. Previously, CCL25 was found to be overexpressed in diverse cancer types and linked to increased hyperplasia and tumor aggressiveness [42]. In our study, using network analysis, we identified CCL25 as key gene interacting with circ_0000069 in HCC. Ding et al. found that CCL25 is a key regulatory gene in miRNA-mRNA regulatory network analysis in non-cirrhotic HCC and cirrhotic HCC [43]. This result is consistent with our results. Typically, CCL25 is involved in T-cell recruitment to inflammation sites, but its cancer-related role varies with the tumor microenvironment [44].The CCR9/CCL25 signaling has also been suggested to positively influence the aggressiveness of HCC cells by affecting epithelial-mesenchymal transition markers, pointing to a potential therapeutic target to mitigate HCC metastasis [45]. In our experiments, silencing CCL25 mitigated the tumorigenic effects of hsa_circ_0000069 overexpression in vivo, proposing CCL25 as a potential negative regulator in HCC. These findings are consistent with previous studies, enhancing the reliability of our results.

Additionally, MAP2K1, affected by hsa_circ_0000069, encodes MEK1-a kinase integral to the MAPK/ERK signaling pathway, which regulates cell division, differentiation, and migration [46]. Mutations in MAP2K1 have been linked to poor prognosis in several cancers, including non-small cell lung, papillary thyroid, and colorectal cancers [47, 48]. A study demonstrated that the acceleration of hyperplasia, metastatic ability, and invasiveness of HCC cells was facilitated by MAP2K1 [49]. Furthermore, miR-330-3p inhibits the metastatic ability of HCC cells by targeting MAP2K1 [50]. Additionally, upregulation of MAP2K1, potentially mediated by circRNA ZFR, was observed to promote HCC cell proliferation. Consequently, the dysregulation of MAP2K1 by hsa_circ_0000069 may lead to the pathological activation of the MAPK/ERK pathway, contributing to HCC development and progression.

Our investigation acknowledges several limitations. The qRT-PCR validation was conducted on a modest cohort; thus, a larger sample set is warranted in subsequent studies to reinforce the results. Furthermore, the functional dynamics among circ_0000069, CCL25, and MAP2K1, along with their downstream effectors, remain to be fully elucidated to understand their collective impact on HCC pathogenesis. While our research comprised in vitro and in vivo analyses, clinical trials are crucial to substantiate the diagnostic and therapeutic potential of these molecular markers in HCC. Lastly, given the multifaceted and heterogenous nature of HCC, additional factors may influence its progression and metastasis. Future research should incorporate a broader scope to examine other elements that may interplay with circ_0000069, CCL25, and MAP2K1 in the disease continuum.

## Conclusion

In summary, this study used bioinformatics to find dysregulated genes in HCC tissues. We found that circ_0000069 is upregulated in HCC and may play an oncogenic role by affecting CCL25. Our experiments showed that these interactions significantly impact the migratory and invasive behavior of HCC cells, contributing to tumor progression in vivo. These findings enhance our understanding of the complex molecular processes driving HCC advancement and pinpoint circ_0000069 as a potential target for therapy.

### Supplementary Information

Supplementary Material 1.

Supplementary Material 2.

## Data Availability

TCGA and GSE97332 datasets are available on GDC (Genomic Data Common; https://portal.gdc.cancer.gov/) and GEO (Gene Expression Omnibus; https://www.ncbi.nlm.nih.gov/geo/) data portal.
