# Supplementary material for: Circ_0000069 promotes the development of hepatocellular carcinoma by regulating CCL25
Source: BMC Cancer. 2024 Jul 11;24:827. doi: 10.1186/s12885-024-12594-y (PMC11238365; doi:10.1186/s12885-024-12594-y)
Supplement: Supplementary file 1 — Supplementary Material 1. [file 12885_2024_12594_MOESM1_ESM.pdf]

Original, full-length gel and blot images

Figure 6A

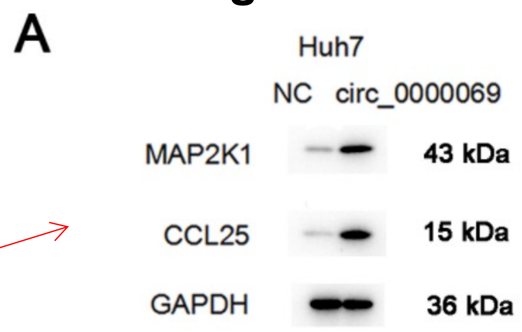

Original

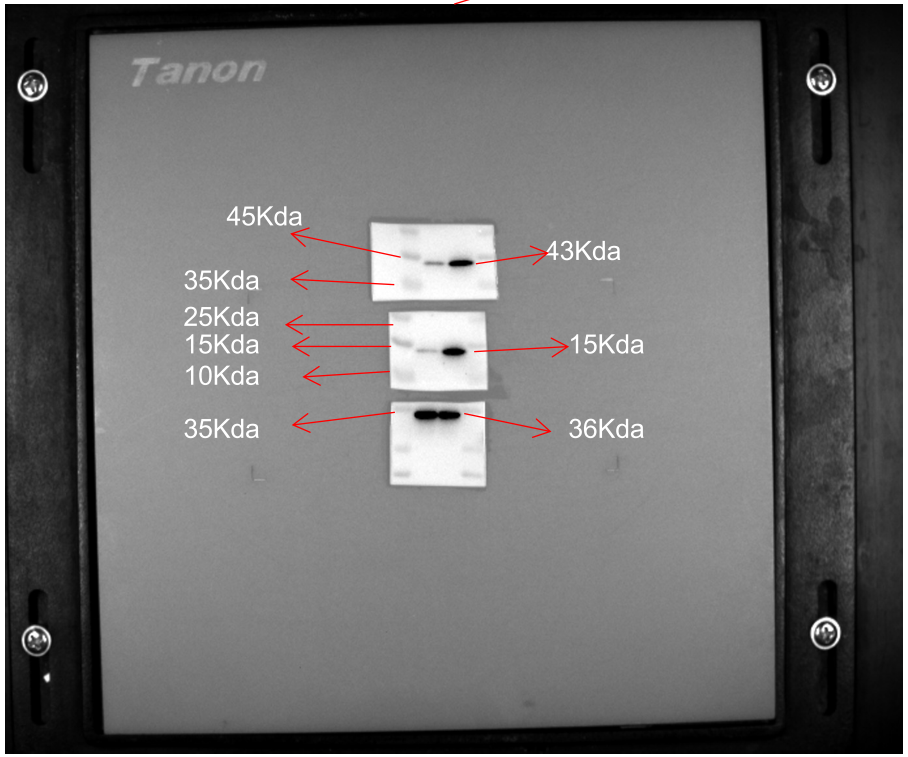

Re-verify

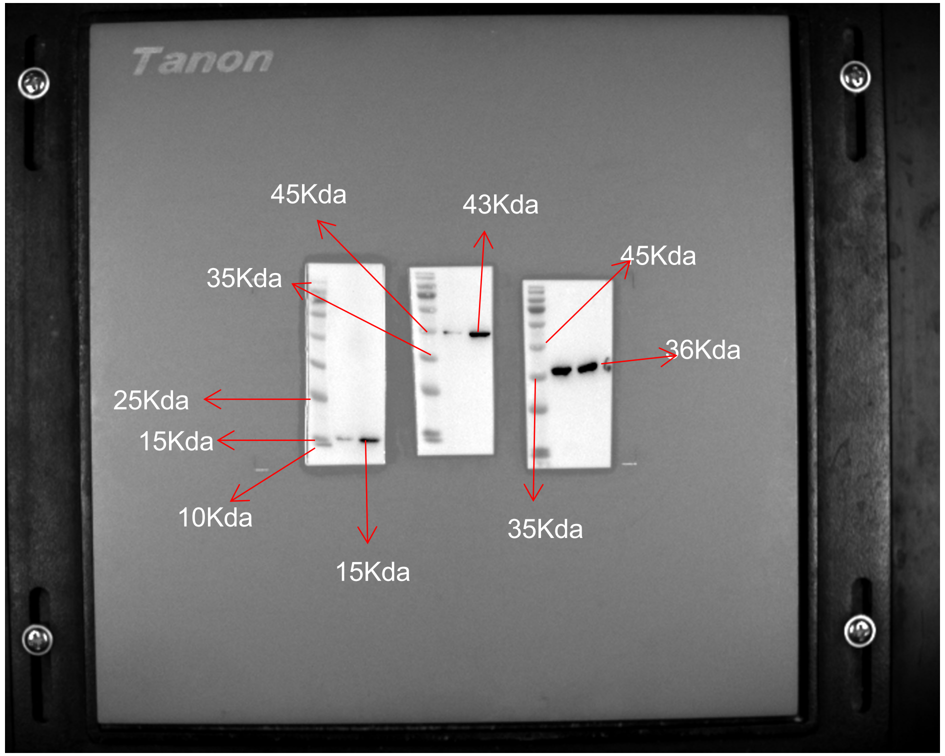

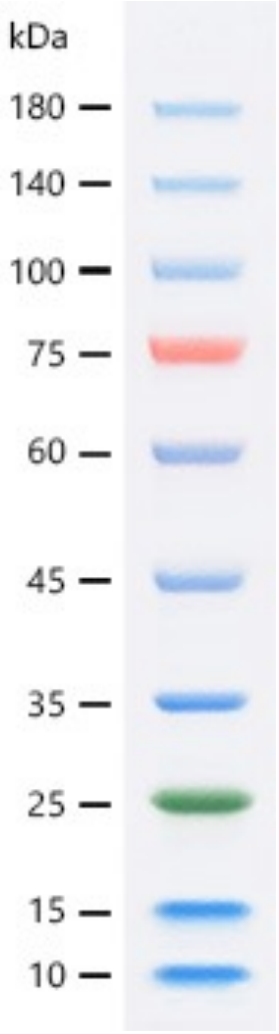

Figure 6C

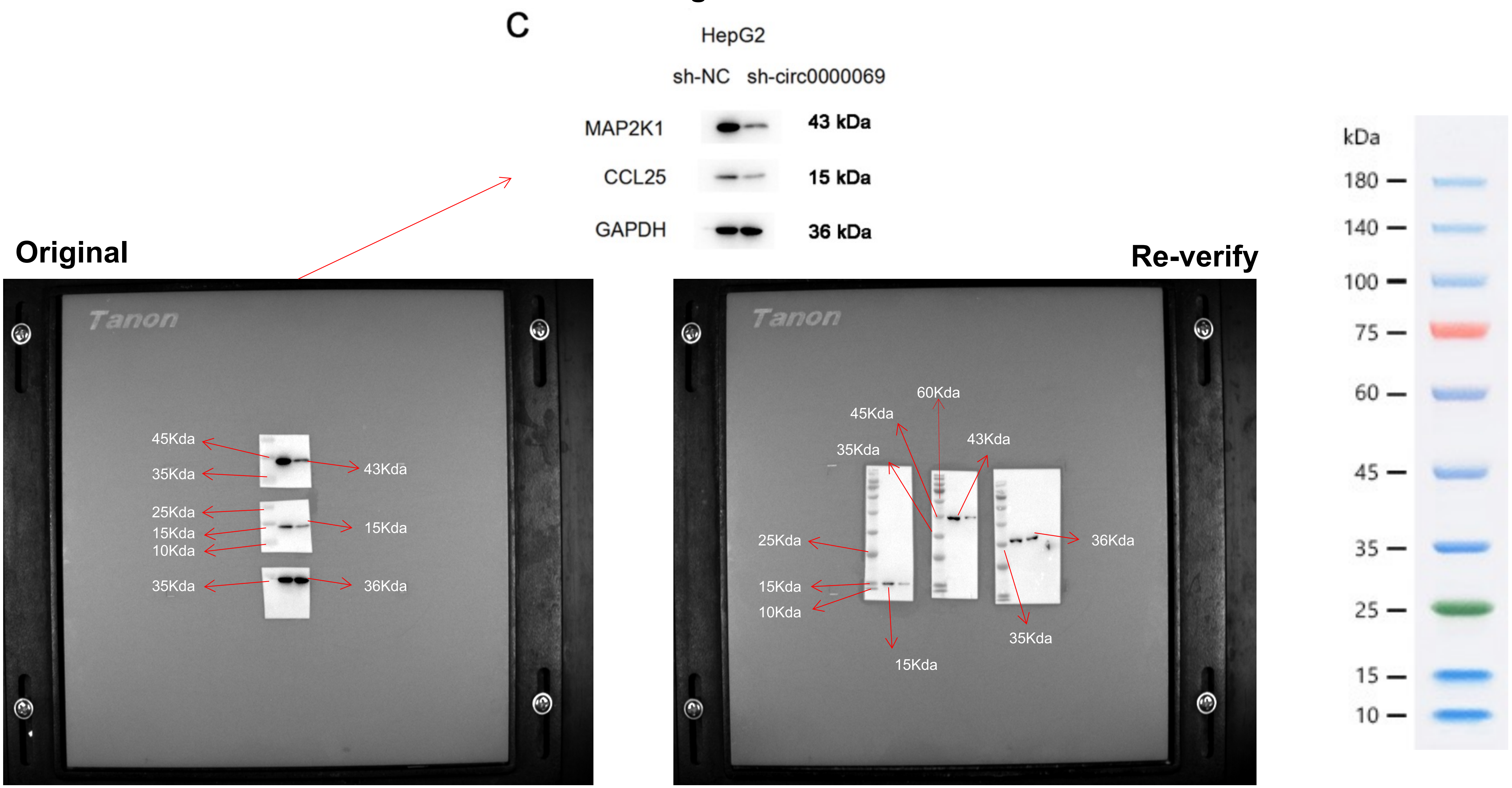
C
HepG2
sh-NC
sh-circ0000069
MAP2K1
43 kDa
CCL25
15 kDa
GAPDH
36 kDa
Original
Tanon
45Kda
35Kda
43Kda
25Kda
15Kda
10Kda
15Kda
35Kda
36Kda
Re-verify
Tanon
60Kda
45Kda
43Kda
35Kda
25Kda
36Kda
15Kda
10Kda
35Kda
15Kda
kDa
180
140
100
75
60
45
35
25
15
10

Figure 6E

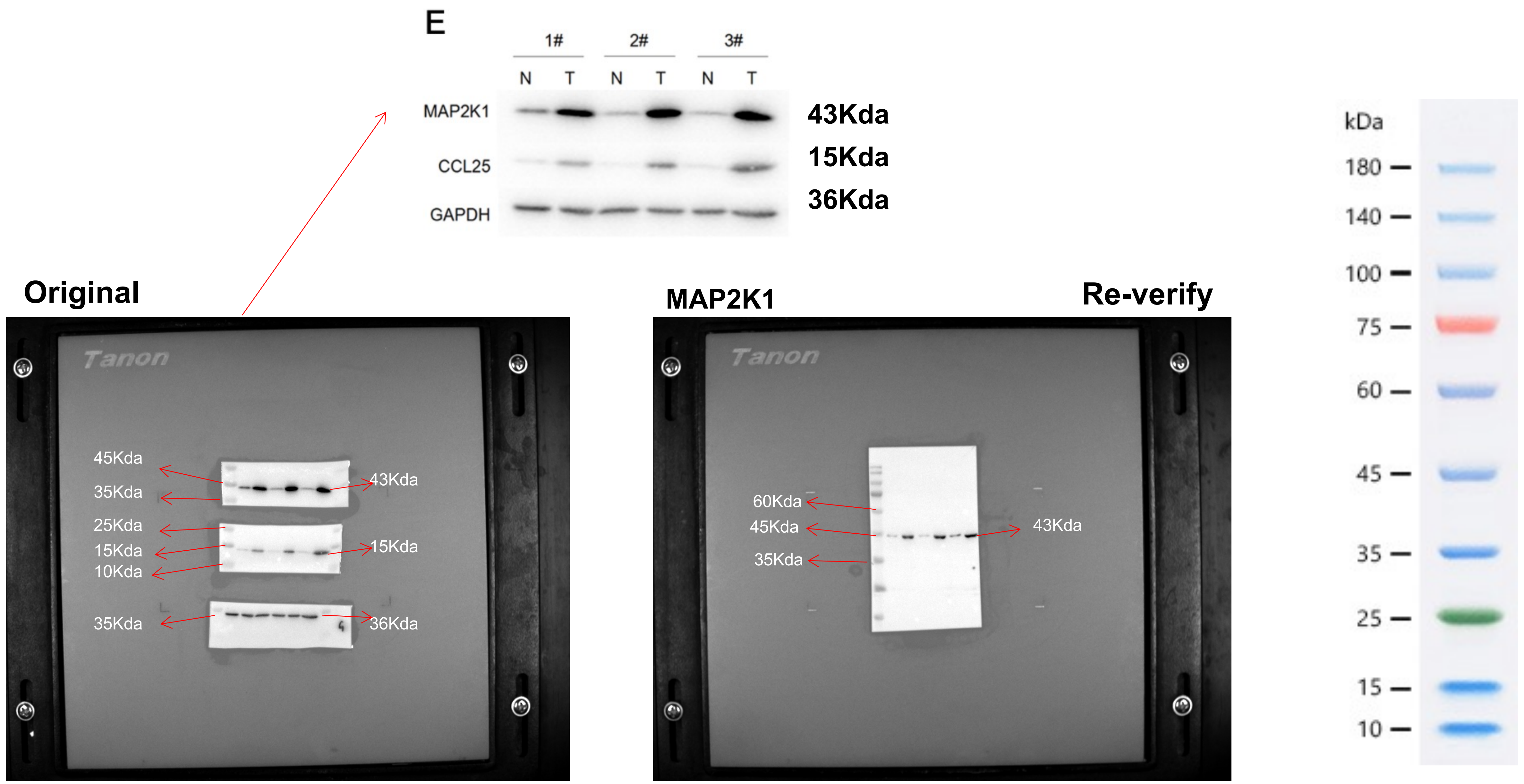

E
1#
2#
3#
N T N T N T
MAP2K1
CCL25
GAPDH
43Kda
15Kda
36Kda
Original
45Kda
35Kda
25Kda
15Kda
10Kda
35Kda
43Kda
15Kda
36Kda
Tanon
MAP2K1
Re-verify
60Kda
45Kda
35Kda
43Kda
Tanon
kDa
180
140
100
75
60
45
35
25
15
10

Figure 6E

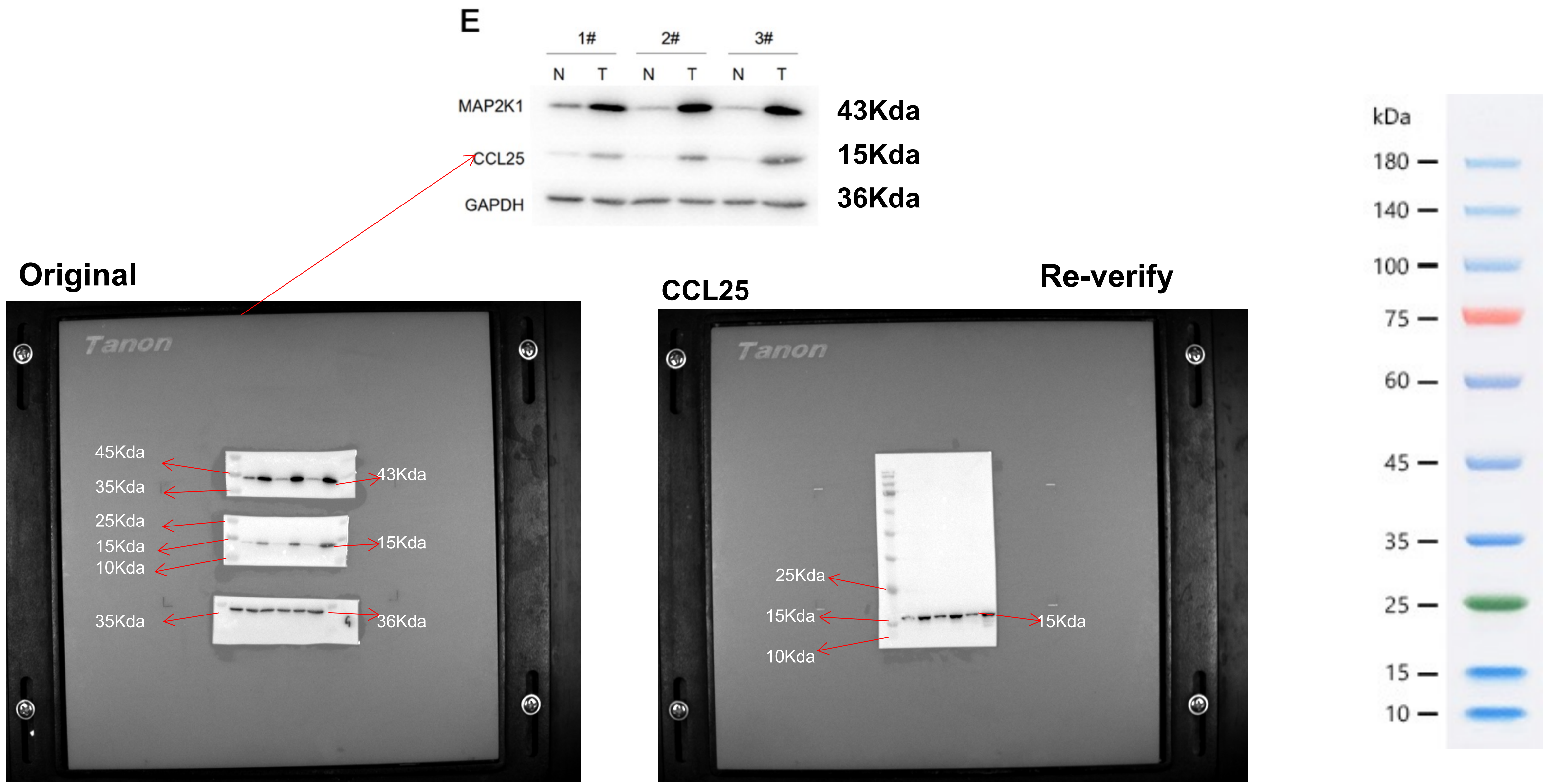
E
1#
2#
3#
N T N T N T
MAP2K1
CCL25
GAPDH
43Kda
15Kda
36Kda
Original
Tanon
45Kda
35Kda
43Kda
25Kda
15Kda
10Kda
15Kda
35Kda
36Kda
CCL25
Re-verify
Tanon
25Kda
15Kda
10Kda
15Kda
kDa
180
140
100
75
60
45
35
25
15
10

Figure 6E

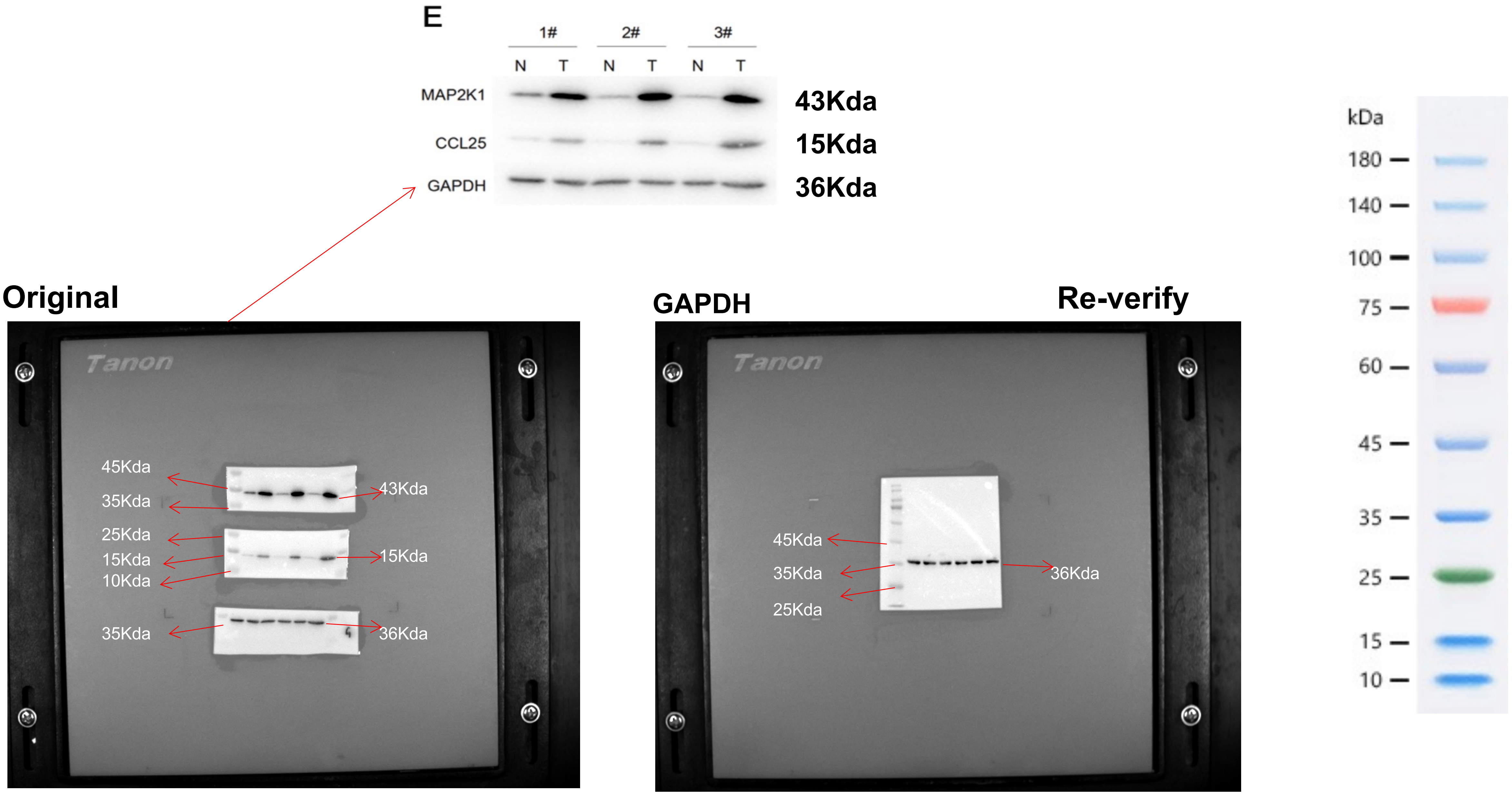
E
1#
2#
3#
N T N T N T
MAP2K1
CCL25
GAPDH
43Kda
15Kda
36Kda
Original
Tanon
45Kda
35Kda
25Kda
15Kda
10Kda
35Kda
43Kda
15Kda
36Kda
GAPDH
Re-verify
Tanon
45Kda
35Kda
25Kda
36Kda
kDa
180
140
100
75
60
45
35
25
15
10
