# Supplementary material for: Circ_0000069 promotes the development of hepatocellular carcinoma by regulating CCL25
Source: BMC Cancer. 2024 Jul 11;24:827. doi: 10.1186/s12885-024-12594-y (PMC11238365; doi:10.1186/s12885-024-12594-y)
Supplement: Supplementary file 2 — Supplementary Material 2. [file 12885_2024_12594_MOESM2_ESM.docx]

Table S1. The results of up-regulated genes in HCC

| **Gene_Name** | **log2FC** | **p_value** | **q_value** | **normal** | **tumor** |
| --- | --- | --- | --- | --- | --- |
| GPC3 | 4.680 | 8.63E-24 | 5.35E-22 | 2.902 | 7.582 |
| AKR1B10 | 3.272 | 3.29E-10 | 3.84E-09 | 3.443 | 6.715 |
| TOP2A | 3.169 | 4.56E-39 | 1.29E-36 | 1.591 | 4.760 |
| MDK | 3.127 | 2.48E-28 | 2.56E-26 | 3.223 | 6.349 |
| MUC13 | 2.870 | 9.80E-11 | 1.22E-09 | 1.413 | 4.283 |

Table S2. The results of up-regulated miRNAs in HCC

| **Gene_Name** | **log2FC** | **p_value** | **q_value** | **normal** | **tumor** |
| --- | --- | --- | --- | --- | --- |
| hsa-miR-10b-5p | 3.209 | 4.50E-27 | 1.67E-25 | 10.238 | 13.447 |
| hsa-miR-224-5p | 2.904 | 5.25E-23 | 9.09E-22 | 3.723 | 6.627 |
| hsa-miR-183-5p | 2.611 | 3.78E-13 | 1.89E-12 | 7.805 | 10.416 |
| hsa-miR-182-5p | 2.287 | 5.02E-12 | 2.04E-11 | 9.717 | 12.004 |
| hsa-miR-452-5p | 2.244 | 3.49E-19 | 3.49E-18 | 5.216 | 7.459 |

Table S3. The partial results of up-regulated circRNAs in HCC

| **Gene_Name** | **log2FC** | **p_value** | **q_value** | **normal** | **tumor** |
| --- | --- | --- | --- | --- | --- |
| hsa_circ_0072088 | 4.565768708 | 7.10E-08 | 3.52E-06 | 10.555 | 15.121 |
| hsa_circ_0046600 | 4.191319866 | 5.25E-13 | 3.64E-10 | 6.173 | 10.364 |
| hsa_circ_0046599 | 3.988266129 | 1.67E-14 | 2.90E-11 | 6.000 | 9.989 |
| hsa_circ_0001955 | 3.815802447 | 6.64E-05 | 2.99E-04 | 11.118 | 14.934 |
| hsa_circ_0005397 | 3.717374059 | 2.27E-07 | 6.92E-06 | 11.149 | 14.866 |

Table S4. The partial up-regulated mRNAs retrieved from the immPort database

| **mRNA** | **synonyms** | **Category** |
| --- | --- | --- |
| KLRD1 | CD94 | NaturalKiller_Cell_Cytotoxicity |
| MAP2K1 | CFC3\|MAPKK1\|MEK1\|MKK1\|PRKMK1 | NaturalKiller_Cell_Cytotoxicity |
| FCGR3A | CD16\|CD16A\|FCG3\|FCGR3\|FCGRIII\|FCR-10\|FCRIII\|FCRIIIA\|IGFR3\|IMD20 | NaturalKiller_Cell_Cytotoxicity |
| FCGR3B | CD16\|CD16A\|CD16b\|FCG3\|FCGR3\|FCGR3A\|FCR-10\|FCRIII\|FCRIIIb | NaturalKiller_Cell_Cytotoxicity |
| FYN | SLK\|SYN\|p59-FYN | NaturalKiller_Cell_Cytotoxicity |

Table S5. The partial pairs between up-regulated miRNAs and down-regulated genes

| **miRNA** | **Target_Gene** | **miRDB** | **TargetScan** | **miRanda** | **miRMap** | **miTarBase** | **Count** |
| --- | --- | --- | --- | --- | --- | --- | --- |
| hsa-miR-10b-5p | RORA | 1 | 1 | 1 | 1 | 1 | 5 |
| hsa-miR-130b-3p | IGF1 | 1 | 1 | 1 | 1 | 1 | 5 |
| hsa-miR-130b-3p | ESR1 | 1 | 1 | 1 | 1 | 1 | 5 |
| hsa-miR-18a-5p | RORA | 1 | 1 | 1 | 1 | 1 | 5 |
| hsa-miR-18a-5p | ESR1 | 1 | 1 | 1 | 1 | 1 | 5 |

(Note: The number 1 represents the miRNAs and target gene have relationship while the number 0 represents they have no relationship.)

Table S6. The partial pairs between down-regulated miRNAs and up-regulated genes

| **miRNA** | **Target_Gene** | **miRDB** | **TargetScan** | **miRanda** | **miRMap** | **miTarBase** | **Count** |
| --- | --- | --- | --- | --- | --- | --- | --- |
| hsa-miR-30a-3p | TNFSF13B | 1 | 1 | 1 | 1 | 1 | 5 |
| hsa-miR-30e-3p | TNFSF13B | 1 | 1 | 1 | 1 | 1 | 5 |
| hsa-miR-142-5p | ROBO1 | 1 | 0 | 1 | 1 | 1 | 4 |
| hsa-miR-195-5p | APLN | 1 | 1 | 1 | 1 | 0 | 4 |
| hsa-miR-29c-3p | ROBO1 | 1 | 1 | 1 | 1 | 0 | 4 |

(Note: The number 1 represents the miRNAs and target genes have relationship while the number 0 represents they have no relationship.)

Table S7. The partial pairs between up-regulated miRNAs and down-regulated circRNAs

| **mirna** | **circrna** |
| --- | --- |
| hsa-miR-103a-3p | hsa_circ_0001495 |
| hsa-miR-103a-3p | hsa_circ_0001861 |
| hsa-miR-103a-3p | hsa_circ_0000391 |
| hsa-miR-10b-5p | hsa_circ_0000045 |
| hsa-miR-1301-3p | hsa_circ_0000045 |

Table S8. The partial pairs between down-regulated miRNAs and up-regulated circRNAs

| **mirna** | **circrna** |
| --- | --- |
| hsa-miR-101-3p | hsa_circ_0000069 |
| hsa-miR-101-3p | hsa_circ_0000727 |
| hsa-miR-122-5p | hsa_circ_0000116 |
| hsa-miR-125b-5p | hsa_circ_0000069 |
| hsa-miR-125b-5p | hsa_circ_0000688 |
